# Generation, Characterization and Epitope Mapping of Two Neutralizing and Protective Human Recombinant Antibodies against Influenza A H5N1 Viruses

**DOI:** 10.1371/journal.pone.0005476

**Published:** 2009-05-07

**Authors:** Lina Sun, Xiuhua Lu, Chuan Li, Min Wang, Qinzhi Liu, Zi Li, Xiaofen Hu, Jiandong Li, Feng Liu, Qun Li, Jessica A. Belser, Kathy Hancock, Yuelong Shu, Jacqueline M. Katz, Mifang Liang, Dexin Li

**Affiliations:** 1 State Key Laboratory for Infectious Disease Control and Prevention, National Institute for Viral Disease Control and Prevention, China CDC, Beijing, China; 2 Influenza Division, National Center for Immunization and Respiratory Diseases, Centers for Disease Control and Prevention, Atlanta, Georgia, United States of America; 3 State Key Laboratory for Molecular Virology and Genetic Engineering, National Institute for Viral Disease Control and Prevention, China CDC, Beijing, China; 4 Anhui Provincial Center for Disease Control and Prevention, Heifei, China; University of Toronto, Canada

## Abstract

**Background:**

The development of new therapeutic targets and strategies to control highly pathogenic avian influenza (HPAI) H5N1 virus infection in humans is urgently needed. Broadly cross-neutralizing recombinant human antibodies obtained from the survivors of H5N1 avian influenza provide an important role in immunotherapy for human H5N1 virus infection and definition of the critical epitopes for vaccine development.

**Methodology/Principal Findings:**

We have characterized two recombinant baculovirus-expressed human antibodies (rhAbs), AVFluIgG01 and AVFluIgG03, generated by screening a Fab antibody phage library derived from a patient recovered from infection with a highly pathogenic avian influenza A H5N1 clade 2.3 virus. AVFluIgG01 cross-neutralized the most of clade 0, clade 1, and clade 2 viruses tested, in contrast, AVFluIgG03 only neutralized clade 2 viruses. Passive immunization of mice with either AVFluIgG01 or AVFluIgG03 antibody resulted in protection from a lethal H5N1 clade 2.3 virus infection. Furthermore, through epitope mapping, we identify two distinct epitopes on H5 HA molecule recognized by these rhAbs and demonstrate their potential to protect against a lethal H5N1 virus infection in a mouse model.

**Conclusions/Significance:**

Importantly, localization of the epitopes recognized by these two neutralizing and protective antibodies has provided, for the first time, insight into the human antibody responses to H5N1 viruses which contribute to the H5 immunity in the recovered patient. These results highlight the potential of a rhAbs treatment strategy for human H5N1 virus infection and provide new insight for the development of effective H5N1 pandemic vaccines.

## Introduction

Multiple distinct and geographically diverse genotypes of highly pathogenic avian influenza (HPAI) A H5N1 viruses now exist and continue to cause outbreaks of disease in domestic poultry on three continents [1], [2]. The occasional spill-over of HPAI H5N1 virus into humans has, since late 2003, resulted in over 387 confirmed human cases of H5N1 influenza of which 245 have been fatal [3]. H5N1 viruses are now endemic in multiple countries in parts of Asia, Africa, and possibly the Middle East [2]. Accordingly, these viruses pose a substantial public health threat; if H5N1 viruses acquire the ability to spread efficiently in humans lacking antibody-mediated immunity to the H5 surface protein, a pandemic would result. If the virus retains its current virulence for humans, an H5N1 pandemic would have catastrophic consequences.

Influenza A viruses are enveloped RNA viruses in the family Orthomyxoviridae possessing eight negative-sense genomic segments and are classified into subtypes based on their two surface glycoproteins, the hemagglutinin (HA) and neuraminidase (NA). There are 16 known HA and 9 NA subtypes that exist in aquatic birds, the natural reservoir of all influenza A viruses [4], [5], [6]. Currently circulating HPAI H5N1 viruses arose from a progenitor virus isolated in China in 1996 [7]. Since 1997, ten distinct clades (0-9) of H5N1 viruses have been recognized based on the phylogeny of the H5 HA gene [7]. Clade 0 viruses caused the 1997 Hong Kong outbreak of human disease, whereas the human cases associated with the reemergence of H5N1 viruses in Southeast Asia in 2003–2005 were a result of infection with Clade1 viruses. H5N1 Clade 2.1 viruses are now endemic in Indonesia, whereas Clade 2.2 viruses spread from Qinghai Lake, China in 2005, and are now found in birds in Western Asia, the Middle East, Europe and Africa and have caused fatal human disease in these respective regions. Clade 2.3 H5N1 viruses have played a dominant role in outbreaks in China and adjacent countries in 2005–2007 [2], [8], [9] and have resulted in recent human fatalities in Vietnam and Laos [2], [3]. The multiple clades and subclades of H5N1 viruses causing human disease are also antigenically distinguishable, which poses a considerable problem for H5N1 human vaccine development, since influenza vaccines offer optimal protection when the vaccine strain is a close antigenic match with the circulation virus causing disease [10], [11], [12]. Moreover, treatment options for H5N1 virus-infected patients remain limited and empirical, and resistance of newly emergent H5N1 viruses to either of the two classes of licensed influenza antiviral drugs, further hampers effective treatment [9], [13], [14]. Therefore, the development of new therapeutic targets and strategies to control HPAI H5N1 virus infection in humans is urgently needed.

Neutralizing antibodies directed against the HA glycoprotein are the primary mediator of protection against influenza virus infection [15], [16]. Three HA monomers, each consisting of an HA1 and an HA2 subunit, form the trimeric HA spike protruding from the viral membrane. The HA1 subunit contains the receptor-binding site which mediates viral attachment to the cell membrane, whereas, the HA2 subunit contributes to membrane fusion [17], [18]. Passive immunization with human monoclonal antibodies (mAbs), humanized mouse mAbs or equine F(ab′)_2_ fragments specific for HA has been reported to be effective in protecting animals from death from influenza, even when administrated after H5N1 virus infection [19], [20], [21]. Indeed, there is some evidence that passive immunotherapy may be suitable treatment option for patients with H5N1 virus infection, suggesting that the development of human monoclonal or polyclonal antibodies for such treatment is warranted [22]. Although neutralizing mAbs derived from H5N1 patients have been reported recently [21], [23], the precise epitopes recognized by such antibodies conferring protective immunity against H5N1 viruses are yet to be identified [24].

The structure of influenza virus HA and location of antibody-binding epitopes were first characterized for HA of the human H3 subtype [25]. The H3 three-dimensional structure was used to map antigenic sites on the H1 [26] and H2 [27], and North American H5 HA molecules [28]. To date, three antigenic sites on the H5 HA molecule have been mapped in detail by locating substitutions detected in anti-HA mouse mAb escape mutants of H5N2 or H5N1 viruses on the crystallographic structure of HA [29], [30]. However, epitopes on H5N1 HA recognized by human mAbs are yet to be identified.

In this report, we describe for the first time the generation and characterization of two broadly cross-neutralizing recombinant human antibodies (rhAbs; AVFluIgG01 and AVFluIgG03) generated by screening a Fab antibody phage library derived from a patient recovered from infection with a clade 2.3 H5N1 virus. Furthermore, through epitope mapping, we identify two distinct epitopes on H5 HA molecule recognized by these rhAbs and demonstrate their potential to protect against a lethal H5N1 virus infection in a mouse model. These results highlight the potential of a rhAbs treatment strategy for human H5N1 virus infection and provide new insight for the development of effective H5N1 pandemic vaccines.

## Results

### Generation of two recombinant human antibodies against H5N1 viruses

A combinatorial antibody library, prepared from a 26 year old donor who was infected with H5N1 virus 14 weeks earlier, was screened with purified AH/1/05 (clade 2.3) virus. After four rounds of panning, 43 human Fab clones were selected which demonstrated reactivity with AH/1/05 purified virus by ELISA. Sequence analysis of all 43 selected Fab clones revealed the presence of only two unique clones, both of them comprising an IgG1 Fd heavy chains and lambda light chains. As shown in Table 1, a Fab antibody designated AVFluFab01 represented 18 Fab clones that possessed a unique V_H4_ and V_L2_ sequences, while the antibody designated AVFluFab03 represented the other 25 Fab clones that possessed distinct V_H3_, and V_L1_ sequences. To further characterize the two Fabs, the two unique Fab clones were converted into intact human IgG1 antibodies, AVFluIgG01 and AVFluIgG03.

**Table 1:** Amino acid sequences of variable regions in the H and L chains of H5N1 viruse-specificn Fabs

Fabs	VR^a^	CDR1^b^	CDR2	CDR3
**AVFluFab01**	V_H_	GYYWS	YLFDSGSTNYNPSLTS	RFWGLDGFDI
	V_L_	TGTSSDVGDYNYVS	DVNKRPS	SSYTSSSTWVF
**AVFluFab03**	V_H_	DYAMS	AISGNGGSSTYYADSVKG	DDSYDGGGQYGLHNWFDS
	V_L_	TGSSSNIGAGYDVH	GNSNRPS	QSYDSSLVVF

a VR, variable region; V_H_, heavy chain in VR; V_L_, light chain in VR.

b CDR, complementarity determining region.

### Characterization of AVFluIgG01 and AVFluIgG03 *in vitro* and *in vivo*

The AVFluIgG01 and AVFluIgG03 binding properties were characterized using indirect immunofloresence assay (IFA), micro-neutralization assay (MN), and hemagglutination inhibition assay (HI) assays, and *in vivo* by passive immunization study in BALB/c mice.

**Figure 1:**
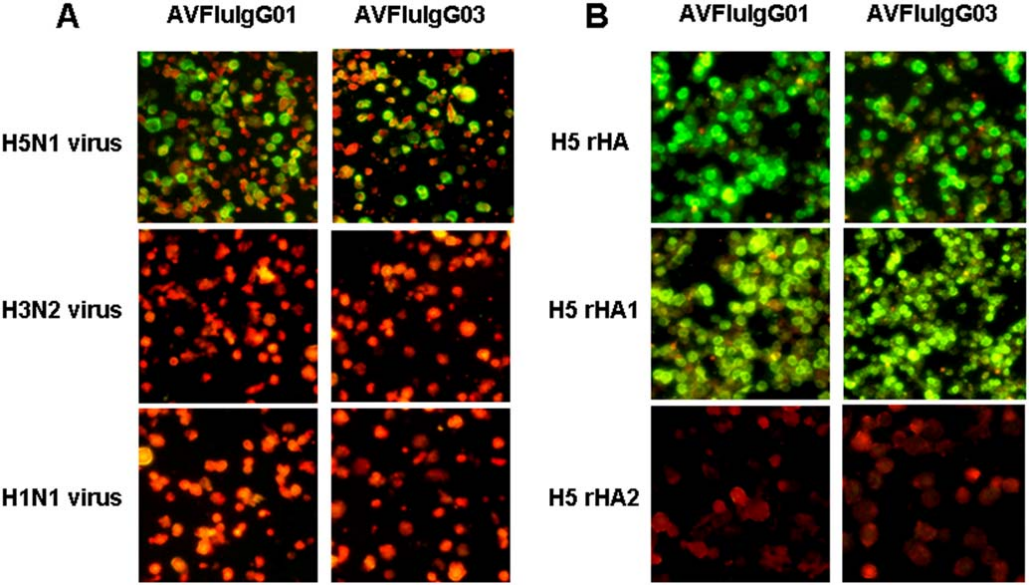
Characterization of AVFluIgG01 and AVFluIgG03 in IFAs. (A) MDCK cells were infected with AH/1/05 (H5N1), HB/53/05 (H1N1), or YN/1145/05 (H3N2) viruses. (B) SF-9 cells were infected with recombinant baculoviruses expressing full length HA, HA1 or HA2 gene from AH/1/05 virus. Bound antibodies were detected by using FITC-conjugated anti-human antibody with PBS dilution buffer (pH 7.4) containing 0.01% (w/v) Evens blue counterstain (Sigma, USA).

To further identified the relative binding specificities and binding regions, the two rhAbs previously shown to bind to AH/1/05 whole virus in ELISA were tested by IFA with MDCK cells infected with H5N1, H3N2, or H1N1 virus (Figure 1A), or SF-9 cells expressing rHA, rHA1 or rHA2 of AH/1/05 virus (Figure 1B). A serum sample from the H5N1 virus-infected patient reacted with H5N1 AH/1/05 and contemporary human influenza A viruses, HB/53/05 (H1N1) and YN/1145/05 (H3N2); and also reacted with rHA, rHA1, and rHA2 of AH/1/05 virus (Supplemental Figure S1). Both AVFluIgG01 and AVFluIgG03 reacted with AH/1/05 virus, but exhibited no reactivity with human H3N2 and H1N1 viruses (Figure 1A). Additionally, both rhAbs reacted with rHA or rHA1 of AH/1/05 virus, but exhibited no reactivity with H5 HA2 region (Figure 1B).

To evaluate the neutralizing activities of the two rhAbs, MN assays were performed first using clade-2.3 and clade-2.2 H5N1 viruses isolated from patients from China (Table 2). With a single exception, AVFluIgG01 showed neutralizing activity against all of the clade 2.3/2.2 viruses tested with the 50% neutralizing antibody concentrations ranging between 1.3–5.2 µg/ml; no AVFluIgG01 neutralizing activity was detected against the clade 2.3 virus, A/Guangdong/1/06 (GD/1/06). In contrast, AVFluIgG03 neutralized all clade 2.3 strains including GD/1/06, and also neutralized the clade 2.2 (XJ/1/06) virus, but required approximately 10-fold more antibody compared with AVFluIgG01 to achieve 50% neutralization. In a second experiment, the rhAbs were tested for their ability to cross-neutralize multiple H5N1 viruses, representing clade 0, 1, 2.1, 2.2 and 2.3 which have all been associated with human disease to date (Table 3). Interestingly, while AVFluIgG01 exhibited broad cross-neutralization of all viruses tested, AVFluIgG03 had no detectable cross-neutralizing activity against the clade 0 and clade 1 viruses, but had neutralizing activity against the all clade 2 viruses tested. The neutralizing activity of the rhAbs against the clade 2.3 virus AH/1/05 were again similar in this experiment, and likewise, the neutralizing activity of AVFluIgG01 against a clade 2.2 virus (Turkey/15/06) was again 10-fold higher than that observed for AVFluIgG03. Taken together, these data demonstrate distinct binding patterns of the two rhAbs for epitope(s) within the HA1 domain of H5N1 viruses.

**Table 2:** Neutralization activity of recombinant human antibodies against influenza A H5N1 viruses isolated from China

Viruses used	Genetic clades	Concentrations (µg/ml)^a^	
		AVFluIgG01	AVFluIgG03
A/Xinjiang/1/06 (XJ/1/06)	2.2	1.3	12.5
A/Anhui/1/05 (AH/1/05)	2.3	1.3	0.8
A/Guangxi/1/05 (GX/1/05)	2.3	2.6	3.1
A/Fujian/1/05 (FJ/1/05)	2.3	2.6	1.6
A/Sichuan/1/06 (SC/1/06)	2.3	5.2	0.8
A/Hunan/1/06 (HN/1/06)	2.3	1.3	0.8
A/Zhejiang/1/06 (ZJ/1/06)	2.3	5.2	3.1
A/Guangdong/1/06 (GD/1/06)	2.3	>500	1.6

**Table 3 pone-0005476-t003:** Neutralization activity of recombinant human antibodies against H5N1 viruses isolated from China and other countries

Viruses used	Genetic clades	Concentrations (µg/ml) a	
		AVFluIgG01	AVFluIgG03
A/HongKong/156/97 (HK/156/97)	0	0.4	>500
A/Viet Nam/1203/04 (VN/1203/04)	1	0.8	>500
A/Indonesia/5/05 (Indo/5/05)	2.1	12.5	6.3
A/Turkey/15/06 (Turkey/15/06)	2.2	0.4	6.3
A/Anhui/1/05 (AH/1/05)	2.3	0.8	0.2

a Minimum concentrations of rhAbs that required to neutralize the 50% infectivity of 100 TCID_50_ of viruses were determined by Micro-neutralization (MN) assay.

To confirm the reactivity patterns of AVFluIgG01 and AVFluIgG03, HI activities against H5N1, H3N2, and H1N1 viruses were assessed (Table 4). The results of the HI assays were consistent with those achieved by the MN assays in that AVFluIgG01 reacted broadly with all H5N1 viruses tested in a concentration range of 1.6–3.1 µg/ml, whereas, AVFluIgG03 reacted in a similar concentration range with all clade 2 viruses, but failed to inhibit hemagglutination of clade 0, and clade 1 H5N1 viruses (Table 3). Lack of binding activity between AVFluIgG03 and rHA of clade 0 and clade 1 H5N1 viruses was also observed in ELISA assays (Supplemental Table S1). Furthermore, the HI assays confirmed the lack of reactivity of either rhAbs for contemporary human influenza A viruses of the H1N1 and H3N2 subtypes.

**Table 4:** HI activity of recombinant human antibodies against influenza A H5N1, H3N2, and H1N1 viruses

Influenza A Viruses used	Subtypes	Genetic clades	Concentrations (µg/ml) a	
			AVFluIgG01	AVFluIgG03
HK/156/97	H5N1	0	1.6	>250
VN/1203/04	H5N1	1	1.6	>250
Indo/5/05	H5N1	2.1	3.1	3.1
Turkey/15/06	H5N1	2.2	1.6	3.1
XJ/1/06	H5N1	2.2	3.1	3.1
AH/1/05	H5N1	2.3	1.6	0.8
GX/1/05	H5N1	2.3	1.6	1.6
FJ/1/05	H5N1	2.3	3.1	1.6
JX/1/05	H5N1	2.3	3.1	1.6
SC/1/06	H5N1	2.3	3.1	1.6
A/Fujian/1/07 (FJ/1/07)	H5N1	2.3	1.6	3.1
A/Wyoming/3/03 (WY/3/03)	H3N2	NA b	>250	>250
A/New Caledonia/20/99 (NC/20/99)	H1N1	NA	>250	>250

a Minimum concentrations, in micrograms per milliliter (µg/ml), that required to completely inhibit 4 HA units of virus were determined by HI assay by using 1% of horse erythrocytes for H5N1 virus or 0.5% of turkey erythrocytes for human H3N2 and H1N1 viruses.

b NA, not applicable.

**Figure 2:**
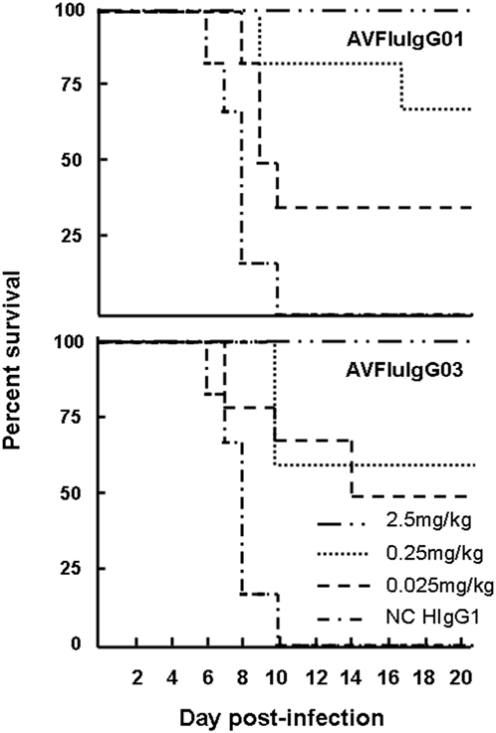
Protective efficacy of AVFluIgG01 and AVFluIgG03 in mice. BALB/c mice (n = 6 per group) were passively immunized by i.p. injection of graded doses of rhAbs, AVFluIgG01 or AVFluIgG03, or human IgG1 (HIgG1) as a negative control (NC). Mice were challenge i.n. with 50 µl of 10 LD_50_ ( = 10^4^MID_50_; 10^5.5^EID_50_) of AH/1/05 virus 24 h later. The data show the Kaplan-Meier survival curves for the 21 days p.i. Mice that received 2.5 mg/kg of either AVFluIgG01 or AVFluIgG03 were completely protected from a lethal challenge (rhAbs versus HIgG1, *p = 0.0006*, log rank test). Both rhAbs also afforded partial protection and delayed days to death at lower doses of 0.25 mg/kg and 0.025 mg/kg (rhAbs versus HIgG1, *p<0.05*, log rank test).

To investigate the protective efficacy of prophylaxis by passive immunization with AVFluIgG01 and AVFluIgG03, BALB/c mice were administered 2.5, 0.25 or 0.025 mg/kg of either rhAb or 10 mg/kg of a control human IgG1 antibody (HIgG1), 24 hr prior to challenge i.n. with 10 LD_50_ ( = 10^4^MID_50_;  = 10^5.5^EID_50_) of AH/1/05 wild-type virus. As shown in Figure 2, all mice receiving the control HIgG1 succumbed to the lethal H5N1 virus by day 10 post-infection (p.i.). In contrast, mice that received prophylaxis with a single 2.5 mg/ml dose of AVFluIgG01 or AVFluIgG03 were completely protected from lethal disease (*p = 0.0006*). A dose-dependent decrease in protective efficacy was observed in mice receiving 10- or 100-fold lower amounts of either rhAb. Although the lower doses of AVFluIgG01 or AVFluIgG03 did not completely prevent fatal disease, they delayed time to death (*p<0.05*). Nevertheless, even at a dose of 0.025 mg/kg, the AVFluIg03 protected 50% of mice from lethal disease (*p = 0.045*). Although lung and brain virus titers determined 6 days p.i. were reduced (≥3-fold) in mice that received 2.5 mg/kg of either rhAb compared with those receiving the control HIgG1, theses differences did not achieve statistical significance (Supplemental Table S2). These results demonstrate that passive immunization of mice with either anti-H5 clade 2.3 rhAb resulted in protection from lethal H5N1 virus disease.

### Epitope mapping

To identify the potential individual amino acids essential for interaction with the two cross-neutralizing human antibodies, amino acid sequences of HA1 region of some human H5N1 isolates used above in the MN and/or HI assays were aligned (Table 5) and analyzed in the context of the respective rhAbs binding reactivity and the antigenic sites previously identified on the H5 HA1 molecule [19], [30], [31]. AVFluIgG01 showed broad cross-neutralizing reactivity to all H5N1 representatives of clade 0, clade 1 and clade 2 viruses except the clade 2.3 virus GD/1/06 (Table 2). Sequence alignment of HA1 proteins identified two amino acid substitutions at position 123 and 183 (S123P and D183N) that were unique to GD/1/06 virus compared with AH/1/05 virus and other clade 2.3 H5N1 viruses tested with which AVFluIgG01 reacted (Table 5). On the other hand, AVFluIgG03 had failed to react with clade-0 and clade-1 viruses by the MN and horse HI assays (Table 3 and Table 4) and also failed to bind clade 0 (HK/156/97) and clade-1 (VN/1203/04) rHA by ELISA (Supplemental Table S1). As illustrated in Table 5, sequence alignment of the HA1 regions identified eight amino acid differences in the clade 0 and clade 1 viruses compared with the clade 2 H5N1 viruses at positions D124S/N, E126D, S129L, Q138L, T140R/K/S, P141S, N155S, and T156A. Therefore, we next investigated whether each of these amino acid positions contributed to the antibody-binding epitopes recognized by AVFluIgG01 and AVFluIgG03, respectively.

**Table 5:** Amino acid changes in HA1 of the H5N1 viruses isolated from humans

Viruses (clade)	HI and/or MN a		Amino acid positions b									
	AVFluIgG01	AVFluIgG03	123	124	126	129	138	140	141	155	156	183
HK/156/97 (0)	+	−	S	N	D	S	L	R	S	S	A	D
VN/1203/04 (1)	+	−	S	S	E	L	Q	K	S	S	T	D
Indo/5/05 (2.1)	+	+	S	D	E	S	L	S	P	S	T	D
Turkey/15/06 (2.2)	+	+	S	D	E	S	Q	R	S	N	A	D
XJ/1/06 (2.2)	+	+	S	D	E	S	Q	R	S	N	T	D
AH/1/05 (2.3)	+	+	S	D	E	S	Q	T	P	N	T	D
GD/1/06 (2.3)	−	+	P	D	E	S	Q	T	P	N	T	N

a “+”, indicates positive binding; “−“, indicates negative binding in HI and/or MN assays.

b Amino acid numbering is based on H5 HA.

To test the impact of the above identified changes of amino acid substitutions identified in natural H5N1 strains, we generated AH/1/05 rHA gene products possessing single amino acid substitutions as identified above (Table 5) and expressed in 293T cells. The ability of the AVFluIgG01 and AVFluIgG03 to bind to the mutated rHA proteins was detected by IFA. As a control for expression, the IFA reactivity of the polyclonal patient serum was also determined for each mutated protein. As shown in Table 6, the mutation S123P resulted in the complete loss of binding of AVFluIgG01 to the expressed rHA product. In contrast, the other substitution that was unique to GD/1/06, D183N, had no effect on the binding of the AVFluIgG01. For AVFluIgG03, mutations at E126D, S129L and N155S abolished or substantially reduced binding of the AVFluIgG03 to the rHA antigen. The remaining substitutions, D124S/N, Q138L, T140K, P141S, and T156A, had no effect on the binding of either AVFluIgG01 or AVFluIgG03 to expressed rHA. The results indicated that the amino acid residue at position 123 was critical for the binding of AVFluIgG01, while Glu^126^, Ser^129^ and Asn^155^ were important for the binding of AVFluIgG03.

**Table 6:** Epitope mapping of rhAbs to a panel of site mutant rHA of AH/1/05

Mutant site a	AVFluIgG01	AVFluIgG03	PC
rHA1 of wild type	+++ b	+++	+++
Q115G	+++	+++	+++
I116H	−	−	+++
I117P	−	−	+++
P118S	−	+++	+++
K119G	+++	+++	+++
S120N	+	+++	+++
S121A	+	+++	+++
W122G	−	−	+++
S123P	−	+++	+++
D124S/N	+++	+++	+++
H125Y	++	+++	+++
E126D	++	−	+++
A127V	+++	+++	+++
S128Y	+++	+++	+++
S129L	+++	−	+++
Q138L	+++	+++	+++
T140K	+++	+++	+++
P141S	+++	+++	+++
K152G	+++	−	+++
K153G	+++	+++	+++
N154G	+++	+++	+++
N155S/G	+++	+/+	+++
T156A/G	+++	+++	+++
D183N	+++	+++	+++

a The amino acid mutant positions are in H5 numbering.

b IFA were performed on 293T cells transfected with mutant rHA constructions. (+) to (+++) indicates the relative intensity of fluorescence.

**Figure 3:**
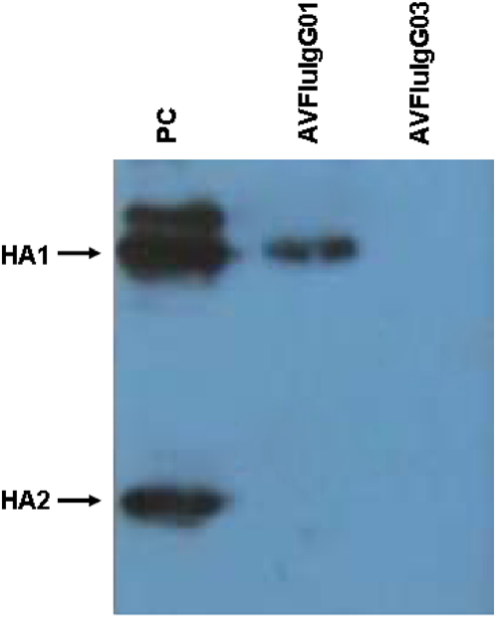
Western Blot analysis of AVFluIgG01 and AVFluIgG03. Purified AH/1/05 virus was applied to SDS–PAGE. The antigens were probed with either AVFluIgG01 or AVFluIgG03. A human serum from H5N1 virus-infected patient was used as a positive control (PC).

**Figure 4:**
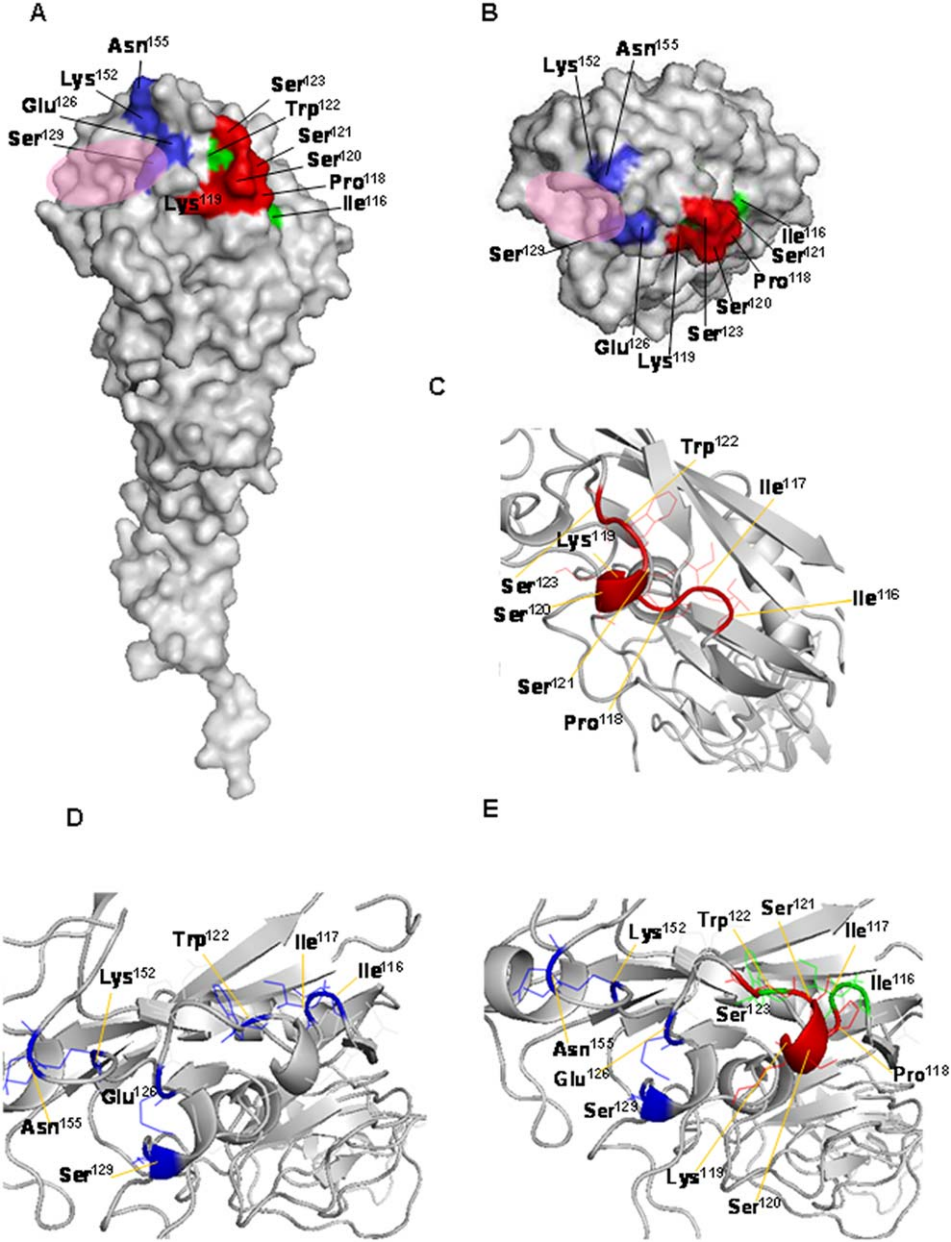
Schematic representation of the epitopes recognized by AVFluIgG01 and AVFluIgG03 on the globular head of the H5 AH/1/05 HA1 molecule. Amino acid positions are designated in H5 numbering. A linear epitope (IIPKSSWS, amino acid 116–123) recognized by AVFluIgG01 is colored in red; non-continuous conformational epitope of AVFluIgG03 is colored in blue; the overlapping binding site for both AVFluIgG01 and AVFluIgG03 are colored in green. The receptor binding domain (RBD) is highlighted with a purple oval. (A) Side view of the HA1 structure. (B) Top view of the globular head. (C) Cartoon illustration of the three-dimensional structure of the linear epitope for AVFluIgG01. (D) Cartoon illustration of the three-dimensional structure of the conformational epitope of AVFluIgG03. (E) Overall structure of the two epitopes of AVFluIgG01 and AVFluIgG03 depicted in cartoon representation.

WB analysis demonstrated that AVFluIgG01 reacted with the denatured viral HA1 protein. In contrast, AVFluIgG03 showed no binding activity with denatured HA1 (Figure 3). The WB result suggested that the epitope recognized by AVFluIgG01 may be a linear epitope, while the epitope recognized by AVFluIgG03 may be a conformational epitope. In an effort to further map the respective epitopes bound by these rhAbs, we generated an additional series of rHA mutants and tested the ability of both rhAbs to bind to the expressed rHA products by IFA. Except for two highly conserved amino acids (Lys^119^ and Trp^122^) each selected amino acid was replaced by a corresponding variable amino acid identified in wild-type human H5N1 virus strains emerging in Asia and Africa respectively (Influenza Virus Resource: http://www.ncbi.nlm.nih.gov/genomes/FLU/FLU.html). The two conserved amino acids were replaced with Glycine (G). To further delineate the binding epitope of AVFluIgG01, single amino acid substitutions around Ser^123^, from HA1 residues 115 to 129 were constructed. Mutations at I116H, I117P, P118S and W122G resulted in complete loss of AVFluIgG01 binding; moreover, mutations at S120N and S121A resulted in substantially reduced binding of AVFluIgG01, but had no impact on the binding of the polyclonal patient's serum (Table 6). In contrast, substitutions at Q115G, K119G, D124S/N, H125Y, E126D, A127V, S128Y and S129L had no or little effect on the binding of this rhAb. These results are consistent with the AVFluIgG01 epitope being linear and comprised of HA1 residues 116–123. Interestingly, the mutations at I116H, I117P, and W122G also led to the abolishment of AVFluIgG03 binding. To further investigate the likely conformational epitope for AVFluIgG03, we analyzed the three-dimensional structure of the H5 HA molecule (Figure 4) and the structure prediction of the H5 A/Anhui/1/2005 HA1 by modeling were created with the software Discovery Studio™ 2.0(Accelrys, USA)and the H5 A/Anhui/1/2005 HA1 structure was obtained from the crystal structure of the highly related the H5 A/Viet Nam/1194/2004 HA1 (PDB accession number 2IBX) by DS MODELER (Discovery Studio 2.0). Amino acid positions are designated in H5 numbering. We noted that amino acids Lys^152^, Lys^153^, Asn^154^, Asn^155^, The^156^, which were on a pocket in the distal part of each HA monomer sterically close to Glu^126^ and Ser^129^. These residues were selected for glycine scanning mutagenesis assay. As shown in Table 6, glycine substitution at K153G, N154G and T156G have little or no effect on the binding for AVFluIgG03. In contrast, the N155G mutations resulted in a noteable reduction in AVFluIgG03 binding, whereas the K152G mutation completely abolished AVFluIgG03 binding, in a manner similar to mutations I116H, I117P, W122G, E126D and S129L. Therefore three non-continuous regions, designated site I (Ile^116^, Ile^117^, Trp^122^), site II (Glu^126^, Ser^129^) and Site III (Lys^152^, Asn^155^) likely contribute to the non-continuous conformational epitope for AVFluIgG03.

## Discussion

In the present study, we generated two H5N1-specific rhAbs (AVFluIgG01 and AVFluIgG03) representing the repertoire of Fab clones recovered from the blood of a convalescent H5N1 virus-infected patient (Table 1 and Figure 1). Interestingly, convalescent plasma from this same patient who had donated the blood for generating hAbs a few weeks earlier was used to transfuse a critically ill patient infected with a genetically similar H5N1 virus; the passive immunotherapy recipient subsequently recovered [32], [33]. Furthermore, as demonstrated in this study, the rhAbs were confirmed to protect mice from lethal H5N1 disease (Figure 2), suggesting that they may represent the dominant B cell response in the recovered H5N1 virus-infected patient. Generation of such human antibodies provides not only important insight into the protective immune response to H5N1 virus in humans, but also provides a valuable treatment option for future H5N1 virus-infected patients [32], [34], [35].

The immune reactivity profile showed that AVFluIgG01 had broad cross HI and/or neutralizing activity *in vitro* against all viruses tested except one clade 2.3 virus, GD/1/06 in which one amino acid substitution (S123P) was implicated in the loss of reactivity with this virus (Table 2–4). On the other hand, AVFluIgG03 showed a more narrow HI and neutralizing profile in that it failed to react with clade 0 and clade 1 viruses, but exhibited strong and broad cross activity for all 2005 and 2006 clade 2 virus strains tested (Table 2, Table 3, Table 4). Both rhAbs gave 50% neutralization of H5N1 viruses in the 0.2–12.5 µg/ml range and protected 100% of mice from fatal disease at a dose of 2.5 mg/kg, doses that were comparable to the virus-neutralizing and lowest in vivo protective concentrations of human H5-specific mAbs derived from memory B cells of a clade 1 H5N1 virus-infected patients reported elsewhere [21]. Although we observed an antibody dose-dependent reduction in viral load in the lung and brain tissue, our results did not achieve statistical significance, at this antibody dose (2.5 mg/kg) that conferred complete protection from death. It should be noted that, where other studies have demonstrated significant reduction in viral load in lung and brain tissues in mice given human H5 antibody prophylaxis, the doses used to demonstrate protection were up to 8 times higher than the dose that gave complete protection from death in our study [21]. We predict that, had we used higher doses of antibody, or a less stringent challenge dose, we would have observed a significant correlation between reduction in viral load and survival. Further studies are needed to address the mechanism of antibody action in protection from death. Our previous studies in mice have suggested that a reduction in extrapulmonary spread is important in survival from H5N1 virus infection [41]. The mouse model used in this study described the primary functionality of the antibodies, we are planning more detailed studies to address the prophylactic or therapeutic potential of these antibodies, either alone or in combination, for their ability to protect from severe disease induced by homologous H5N1 virus, as well as heterologous H5N1 viruses of other clades. We think that it would help to understand the relevance of the epitopes and their mode of action.

The epitopes recognized by AVFluIgG01 or AVFluIgG03 were on the head of the HA1, but not HA2 molecule and appeared to be linear or conformational, respectively (Figure 1 and Figure 3). Sequence alignment and site-directed mutagenesis of HA (AH/1/05) identified amino acid residues that were critical for the binding activity of the two H5-specific rhAbs (Table 5 and Table 6). For the linear epitope recognized by AVFluIgG01, alignment of the H5N1 viruses HA1 used in MN assays revealed that GD/1/06 HA1 differed in 2 amino acids (S123P, D183N) from the wild-type AH/1/05 and/or other clade 2.3 H5N1 viruses, thereby providing important insight as to the location of the linear epitope. The results of site-directed mutagenesis indicated that the linear epitope for AVFluIgG01 was contained within a sequence of IIPKSSWS (amino acid 116–123 of HA1) and identifies for the first time a linear neutralizing antigenic epitope recognized by H5N1 patient-derived human antibody, of which the critical amino acid residues (Ile^116^, Ile^117^, Pro^118^, Trp^122^, Ser^123^) were not reported previously. Interestingly, mutations I116H, I117P and W122G also abolished AVFluIgG03 binding. Furthermore, by examining the positions of the AH/1/05 HA1 sequences on three-dimensional H5 HA structure as illustrated in Figure 4, we observed that this newly defined linear epitope recognized by AVFluIgG01 (colored in red and green) (Figure 4A and 4B) comprises a remarkably tight cluster on the exposed surface of the globular head of the HA1 H5 molecule, forming a ridge that protrudes prominently from the H5 AH/1/05 HA1 surface, that is in close proximity to the receptor binding region (RBD), which comprises 190 helix, 130 loop and 220 loop as colored in pink in Figure 4A and 4B [31], [36]. The position of the surface-exposed epitope is consistent with a direct role of AVFluIgG01 in neutralizing virus infectivity through binding the epitope on the HA spike and block attachment of virus to the receptor. Moreover, by performing a bioinformatics analysis, using the 266 human H5N1 virus strains available in the database of Influenza Virus Resource (http://www.ncbi.nlm.nih.gov/genomes/FLU/FLU.html), we found that residues Lys^119^ and Trp^122^ were absolutely conserved while other residues in the proposed linear epitope were highly conserved among 266 human H5N1 viruses (96.2%–99.9%). Although our results indicated that the single mutation K119G had no direct effect on the antibody binding, the illustration of the three dimensional structure shown in Figure 4C and 4E indicates that residues Lys^119^, Ser^120^ and Ser^121^ comprise a major helix structure with Ser^120^ and Ser^121^ are positioned on the outer face of the helix (96.2% conservation), whereas Lys^119^ is positioned in the inner helix, which may explain the conservation of this residue among 266 human H5N1 viruses. Residue Ile^117^ is partially buried at the surface of the globular head of the HA1 and may contribute through stabilizing and/or positioning of the linear epitope on the globular head of the HA1 H5 molecule. We propose that this novel linear epitope defined by the broadly neutralizing human antibody AVFluIgG01 represents a highly conserved neutralization epitope that may play an important role in inducing protective humoral immune responses to H5N1 viruses in humans. In contrast to the continuous epitope identified by AVFluIgG01, the epitope recognized by AVFluIgG03, is comprised of three non-continuous sites which comprise a conformational determinant as illustrated in blue and green in Figure 4 (A, B, D, and E),from the top view of the H5 three-dimensional structure (Figure 4B and 4E). Site I (Ile^116^, Ile^117^ and Trp^122^) in green, partially overlaps with the linear epitope identified by AVFluIgG01 but is not exposed from the upper surface of the H5 HA molecule, suggesting that this site may not directly interact with the AVFluIgG03 but may otherwise affect the stability of the conformational epitope on the globular head of the HA1 H5 molecule. Site II (Glu^126^ and Ser^129^) overlaps with a previously described antigenic site 3 in H5 HA based on the X-ray crystallographic structure of the clade 1 virus VN/1203/04 virus [30], [31] and is adjacent to the Sa site defined in the H1 subtype based on the antigenic structure analysis of A/Puerto Rico/8/34 virus with mouse mAbs [26]. Site III (Lys^152^, Asn^155^) partially corresponds to the previously identified antigenic site 2 in the H5 HA [29], [30] and site B in the H3 HA structure [25]. Both site II and the site III are exposed on the upper surface of the H5 HA molecule and are located along the upper edge of receptor-binding domain pocket (RBD; in purple) (Figure 4A and 4B). Residues 126, 129 and 155 were initially implicated in the binding of AVFluIgG03 since differences at these positions were identified between the 2005 clade 2.3 AH/1/05 and the clade 0 and clade 1 viruses which failed to react with the AVFluIgG03 by MN and HI tests (Table 2–4). Site-directed mutagenesis of AH/1/05 HA confirmed that the residues Glu^126^ and Ser^129^ were critical residues for the binding of AVFluIgG03. Interestingly, the H5 epitope for the human antibody AVFluIgG03 overlaps with epitopes recognized by mouse mAbs on a mouse-adapted duck H5N2 virus (A/Mallard/Pennsylvania/10218/84) identified by the sequence analysis of virus escape mutants [29], [30]. Using a similar approach, amino acid residue 152 was identified as a key position within the epitope recognized by certain mouse mAbs on the clade 1 H5N1 virus VN/1203/04 HA [30]. These results suggest that the conformational antigenic determinant defined here may be a key region for drift variation among H5N1 viruses that have infected humans.

In summary, we have generated and characterized two recombinant baculovirus-expressed human neutralizing and protective antibodies directed against an H5N1 clade 2.3 virus which exhibit unique properties for intra and inter-clade virus reactivity. Importantly, localization of the epitopes recognized by the two rhAbs has provided, for the first time, insight into the human antibody responses to H5N1 viruses which contribute to the H5 immunity in the recovered patient. The primary prophylactic functionality of the antibodies were addressed in this study with the mouse model. More detailed studies in vivo would help to understand the significance of the defined epitopes and the important mechanisms for prophylaxis or therapy of human infection with H5N1 viruses. The utility of the recombinant approach allows for rapid scale-up in production, as well as a means to rapidly clone and express antibodies with specificity for newly emerging H5N1 variants. More functional human antibodies could be obtained by additional screenings. The rhAbs described here alone or in combination with other functional human antibodies may provide a promising for broadly neutralizing passive immunotherapy treatment that could supplement existing antiviral strategies against human H5N1 virus infection.

## Materials and Methods

### Viruses

Influenza viruses used in this study were propagated at 37°C in the allantoic cavity of 10-day-old embryonated hens' eggs for 26 hours (H5N1 virus) or 48 hours (H3N2 and H1N1 viruses), and were aliquoted and stored at −70°C until use. Fifty percent egg infectious dose (EID_50_) titers were determined by serial titration of virus in eggs and calculated by the method of Reed and Muench [37]. A/Anhui/1/2005 (AH/1/05) virus was propagated in MDCK cells. Culture supernatants were clarified by low-speed centrifugation to remove cell debris, and were further purified by using continuous sucrose density gradient ultracentrifugation.

### Generation of recombinant human antibodies to H5N1 virus

Lymphocytes used for mRNA extraction were isolated from blood that was collected from a 26-year-old female convalescent H5N1 virus-infected patient from Anhui province. The blood donor developed symptoms on 11 February 2006 following contact with diseased poultry [22], [33] and convalescent blood was obtained 14 weeks after the onset of a disease. The written informed consent was agreed by the patient. Total cellular mRNA was extracted and cDNA was synthesized. The heavy and light chain genes were amplified from the cDNAs by PCR and sequentially cloned into the phagemid vector pComb3H. The H5N1 virus-infected patient antibody phage library was constructed by using primers and methods as previously described [38]. The antibody library was screened by panning on purified AH/1/05 virus [39]. After three or four rounds of panning, crude Fab antibody preparations were tested by indirect ELISA using 96 well plates coated with 0.5–1 µg of purified AH/1/05 virus. HRP-conjugated anti-human Fab was used as the secondary antibody. The selected human Fab antibody genes were sequenced and two of them were converted to human IgG by cloning the Fab genes into IgG expression cassette vectors pAc-L-Fc as previously described [40]. The two rhAbs (AVFluIgG01 and AVFluIgG03) were expressed in SF9 cells and purified on a Protein A column for further characterization and functional analysis. Purity of rhAbs was confirmed using SDS-PAGE analysis.

### Construction and expression of rHA, rHA1, and rHA2

The viral RNA was extracted from AH/1/05 virus and the cDNA was synthesised. The DNA fragments encoding full length HA, HA1 or HA2 were amplified by RT-PCR and cloned into pAcUW51 vector (BD Bioscienses) and then expressed in SF-9 insect cells as previously described [41], [42].

### Immunofluorescence Assay (IFA)

IFAs were performed on different cells according to the experimental design. To measure rhAbs reactivity for viral antigens, Madin Darby Canine Kidney (MDCK) cells were infected respectively with AH/1/05 (H5N1), A/Hubeihongshan/53/2005 (HB/53/05; H1N1), and A/Yunnan/1145/2005 (YN/1145/05; H3N2) viruses. To measure rhAbs reactivity for viral recombinant HA (rHA), rHA1, or rHA2, SF-9 cells were infected with recombinant baculoviruses expressing HA, HA1 or HA2 products from AH/1/05 virus, respectively. To evaluate rHA containing site-directed mutations, rHAs were transiently expressed in 293T cells. Cells were grown in 24 wells plates and were then either directly stained in the wells or were prepared as monolayer on glass slides followed by acetone fixation. Bound antibodies were detected by using FITC-conjugated anti-human antibodies and observation under an immunofluorescence microscope.

### Microneutralization assay

To verify the neutralizing ability of AVFluIgG01 and AVFluIgG03, the micro-neutralization (MN) assay was performed as previously described [43]. Neutralizing antibody titers are expressed as the concentrations of rhAbs that gave 50% neutralization of 100 50% tissue culture infectious dose (TCID_50_) of virus in MDCK cells.

### Hemagglutination-inhibition (HI) assay

To verify the HI ability of AVFluIgG01 and AVFluIgG03 against avian H5N1 or human H3N2 and H1N1 viruses, the HI assays were performed using 0.5% turkey red blood cells for detecting reactivity with human H3N2 and H1N1 virus [44] or 1% horse red blood cells for detecting reactivity with avian H5N1 viruses [45]. HI antibody titers are expressed as the concentrations of rhAbs that completely inhibited 4 hemagglutinating units (HAU) of virus.

### Passive immunization of mice and challenge experiment

The fifty percent mouse infectious dose (MID_50_) and 50% lethal dose (LD_50_) of AH/1/05 virus were determined as previously described [46]. To evaluate the degree of protection, 8 week-old female BALB/c mice (Jackson Laboratories, Bar Harbor, MA, USA) were intraperitoneally (i.p.) injected with 0.5 ml of purified rhAb preparations of various concentrations or hyperimmune rabbit antiserum raised against baculovirus expressed H5 rHA of A/Vietnam/1203/2004 virus (VN/1203/04) (Protein Sciences Corporation, Meriden, CT, USA) as a positive control (PC). Negative control (NC) antibody was a purified human myeloma IgG1 (Sigma, Missouri, USA). Twenty-four hours after passive immunization, mice were lightly anesthetized by inhalation of CO_2_ and challenged intranasally (i.n.) with 10 LD_50_ ( = 10^4^MID_50_; 10^5.5^EID_50_) of AH/1/05 virus in 50 µl. Mice were monitored daily for sickness, weight loss and death for 21 days.

### Western blot (WB) analysis

Purified virus of AH/1/05 were applied to 10% SDS–PAGE and transferred to a PVDF membrane with a mini-protean apparatus (Bio-Rad). The denatured antigens were probed with patient sera (1:150 dilution) as a PC or 1 µg/ml of an anti-SARS IgG as a NC or the two rhAbs at 1 µg/ml. A goat anti-human IgG conjugated to HRP was used as the secondary antibody. The reaction was detected by ECL reagent (Pierce) according to the manufacturer's instructions.

### Amino acid substitutions by site-directed mutagenesis

Mutagenic primers were designed after aligning sequences of HAs region of MN and HI tested human H5N1 isolates including A/Anhui/1/2005 (Genebank accession DQ371928), A/Guangxi/1/2005 (DQ371930), A/Hunan/1/2006 (FJ492879), A/Zhejiang/1/2006 (FJ492880), A/Sichuan/1/2006 (FJ492881), A/Fujian/1/2005 (FJ492882), A/Fujian/1/2007 (FJ492883), A/Guangdong/1/2006 (FJ492884), A/Jiangxi/1/05 (FJ492885), A/Xinjiang/1/2006 (FJ492886), A/VietNam/1203/2004 (AY818135), A/HongKong/156/1997 (AF023709), A/Turkey/15/2006 (EF619989) and A/Indonesia/5/2005 (EU146622), and the DNA fragments of site-directed mutagenesis of the HA gene of AH/1/05 were amplified by PCR, cloned into XhoI and BamHI sites of pCDNA3.0, and confirmed by DNA sequencing to exclude secondary mutation. All constructs were transfected to 293T cells for transient expression. The resulting mutated rHAs were assayed by IFA described above for binding activity for AVFluIgG01 and AVFluIgG03.

### Schematic representation of the epitopes recognized by AVFluIgG01 and AVFluIgG03 on the globular head of the H5 HA1 molecule

The H5 AH/1/05 HA1 structure was based on the crystal structure of the highly related the H5 A/Viet Nam/1194/2004 HA1 (PDB accession number 2IBX) by DS MODELER (Discovery Studio 2.0). Images were created with the software Discovery Studio™ 2.0 (Accelrys, USA).

### Statistical analysis

Kaplan-Meier survival curves and logrank tests were used to measure differences between rhAb treated mice and NC mice.

## Supporting Information

Table S1 (0.04 MB DOC)

Table S2 (0.03 MB DOC)

Figure S1 (2.25 MB TIF)
