# Supplementary material for: Generation, Characterization and Epitope Mapping of Two Neutralizing and Protective Human Recombinant Antibodies against Influenza A H5N1 Viruses
Source: PLoS One. 2009 May 7;4(5):e5476. doi: 10.1371/journal.pone.0005476 (PMC2674214; doi:10.1371/journal.pone.0005476)
Supplement: Table S1 — (0.04 MB DOC) [file pone.0005476.s001.doc]

**Table S1. IgG titers of human H5 mAbs against influenza A H5N1, H3N2, and H1N1 rHA or rNA.**

|  |  | Reciprocal IgG titers against indicated influenza A rHA or rNA (log_10_) *^a^* | | | | | | |
| --- | --- | --- | --- | --- | --- | --- | --- | --- |
|  |  | rHA | | | |  | rNA | |
| Antibodies | Concentration | H3N2 WY/03 | H1N1 NC/20/99 | H5N1 HK/156/97 | H5N1 VN/1203/04 |  | H5N1 HK/483/97 | H1N1 BJ/262/95 |
| HIgG1 (Sigma) | 1 mg/ml | <1 | <1 | <1 | <1 |  | <1 | <1 |
| AVFluIgG01 | 1 mg/ml | 2.7 | <1 | 5.7 | 5.8 |  | 1.8 | 1.8 |
| AVFluIgG03 | 1 mg/ml | 2.8 | <1 | 1.1 | 2.1 |  | 1.7 | 1.3 |
| PC *^b^* | Not known | 3.3 | 5.2 | 6.3 | 6.4 |  | 3.8 | 3.6 |
| NC *^b^* | Not known | <2 | <2 | <2 | <2 |  | <2 | <2.1 |

*^a^* Influenza virus-specific IgG antibody were detected by ELISA as previously described (1), except that 1 µg/mlof purified baculovirus-expressed recombinant HA (rHA) or rNA protein from influenza A H3N2, H1N1, or H5N1 virus were used to coat plates.

*^b^* The serum from a rabbit immunized with H5N1 whole viruses was used as a positive control (PC). The serum collected from a naïve rabbit was used as a negative control (NC) .

1. Katz JM, Lu X, Young SA, Galphin JC (1997) Adjuvant activity of the heat-labile enterotoxin

from enterotoxigenic *Escherichia coli* for oral administration of inactivated influenza virus vaccine. J. Infect. Dis. 175,352-63.
