# Supplementary material for: Generation, Characterization and Epitope Mapping of Two Neutralizing and Protective Human Recombinant Antibodies against Influenza A H5N1 Viruses
Source: PLoS One. 2009 May 7;4(5):e5476. doi: 10.1371/journal.pone.0005476 (PMC2674214; doi:10.1371/journal.pone.0005476)
Supplement: Table S2 — (0.03 MB DOC) [file pone.0005476.s002.doc]

**Table S2. Viral replication in mice following passive immunization^a^**

|  |  | **Viral titer (log_10_ EID_50_/ml)** | |
| --- | --- | --- | --- |
| **Fabs** | **Concentration (mg/kg)** | **Lung** | **Brain** |
| AVFluIgG01 | 2.5 | 6.1 ± 0.6 | 1.5 ± 0.9 |
|  | 0.25 | 6.3 ± 0.4 | 2.5 ± 1.7 |
|  | 0.025 | 6.3 ± 0.4 | 1.7 ± 1.1 |
| AVFluIgG03 | 2.5 | 6.0 ± 0.8 | 3.0 ± 2.4 |
|  | 0.25 | 5.8 ± 0.4 | 2.9 ± 1.0 |
|  | 0.025 | 6.6 ± 0.5 | 3.7 ± 1.8 |
| NC HIgG1 |  | 6.6 ± 0.5 | 3.7 ± 2.0 |

^a^ BALB/c mice were passively immunized by i.p. injection of graded

doses of rhAbs, AVFluIgG01 or AVFluIgG03, or human IgG1 (HIgG1) as

a negative control (NC). Mice were challenged i.n. with 10LD_50_ of AH/1/05

virus 24 hours later. Whole lung and brain tissues were collected on

day 6 p.i., homogenized, and titered in eggs. Values are expressed as

the mean titer of 4 mice per group plus standard deviation. The limit of

detection was 10^1.5^ (lung) or 10^0.8^ (brain) EID_50_/ml.
