# Supplementary figures and images for: Generation, Characterization and Epitope Mapping of Two Neutralizing and Protective Human Recombinant Antibodies against Influenza A H5N1 Viruses
Source: PLoS One. 2009 May 7;4(5):e5476. doi: 10.1371/journal.pone.0005476 (PMC2674214; doi:10.1371/journal.pone.0005476)

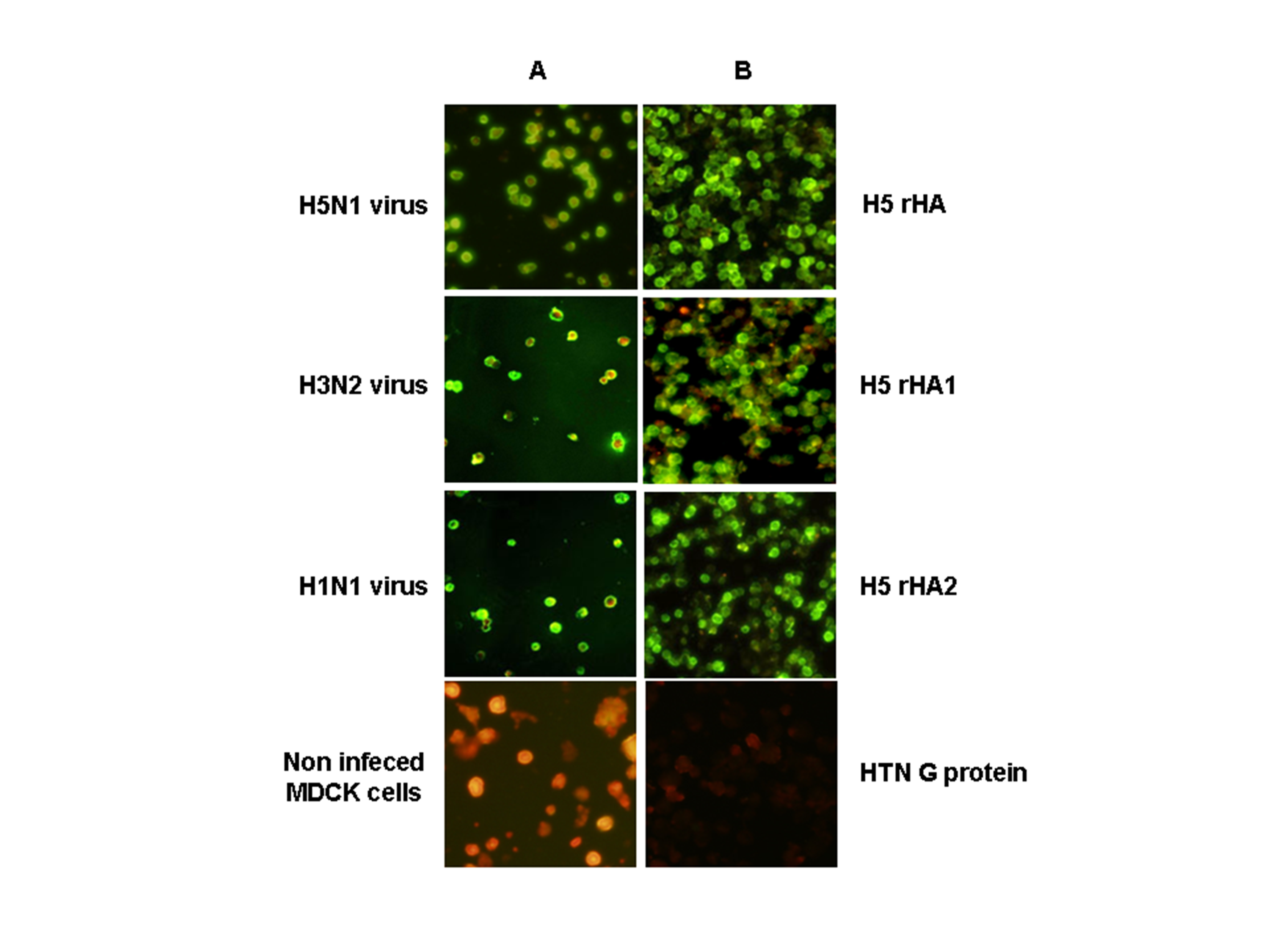

Supplement: Figure S1 — (2.25 MB TIF) [file pone.0005476.s003.tif]
